# High-Throughput Feeding Bioassay for Lepidoptera Larvae

**DOI:** 10.1007/s10886-021-01290-x

**Published:** 2021-07-31

**Authors:** Inoussa Sanané, Judith Legrand, Christine Dillmann, Frédéric Marion-Poll

**Affiliations:** 1 Université Paris-Saclay, INRAE, CNRS, AgroParisTech, GQE - Le Moulon, 91190 Gif-sur-Yvette, France; 2 Université Paris-Saclay, CNRS, IRD, UMR Évolution, Génomes, Comportement et Écologie, 91198 Gif-sur-Yvette, France; 3 Université Paris-Saclay, AgroParisTech, Paris, France

**Keywords:** Plant–insect warfare, Feeding preferences, Digital image analysis, High-throughput device

## Abstract

Finding plant cultivars that are resistant or tolerant against lepidopteran pests, takes time, effort and is costly. We present here a high throughput leaf-disk consumption assay system, to screen plants for resistance or chemicals for their deterrence. A webcam capturing images at regular intervals can follow the feeding activities of 150 larvae placed into individual cages. We developed a computer program running under an open source image analysis program to analyze and measure the surface of each leaf disk over time. We further developed new statistical procedures to analyze the time course of the feeding activities of the larvae and to compare them between treatments. As a test case, we compared how European corn borer larvae respond to a commercial antifeedant containing azadirachtin, and to quinine, which is a bitter alkaloid for many organisms. As expected, increasing doses of azadirachtin reduced and delayed feeding. However, quinine was poorly effective at the range of concentrations tested (10^–5^ M to 10^–2^ M). The model cage, the camera holder, the plugins, and the R scripts are freely available, and can be modified according to the users’ needs.

**Supplementary Information:**

The online version contains supplementary material available at 10.1007/s10886-021-01290-x.

## Introduction

Crops are exposed to increased pressure from insect pests, partly due to climate change which affects the distribution of pest insects (Battisti and Larsson 2015; Castex et al. 2018). The globalization of human activities, which allow pests to cross natural barriers and invade new ecosystems, also contributes to this increase by causing massive direct and indirect economical costs (Bradshaw^2016^). Pests placed in new biotic and abiotic conditions may become invasive, as they lack their usual natural enemies, face plants with poor defenses against them (Sakai^2001^), and may even become more adaptable and colonize new hosts as documented in *Drosophila suzukii* (Little et al. 2020). These changes place plant selection under pressure because it means existing cultivars need to be re-examined for their resistance or tolerance and eventually, new cultivars need to be developed which is both costly and time-consuming. To facilitate the process, high-throughput laboratory assays are needed to identify plants potentially carrying resistance traits, within traditionally bred varieties or material derived from global germplasm (Goggin et al. 2015).

Leaf-disk assays are commonly used to evaluate plant resistance against insects with chewing mouthparts by measuring the area of tissues consumed (Jermy et al. 1968; Jones and Coleman 1988; O'Neal et al. 2002; Sharma et al. 2005). They are also relevant to study stem borers because adult females deposit their eggs on the plant surface, as neonate larvae graze on the leaf surface, and young larvae need to bore a tunnel through the leaves to reach the inner tissues. Leaf-consumption by young larvae can thus be used as a proxy to evaluate plant resistance and to find and evaluate the effectiveness of feeding deterrents extracted from plants or of synthetic origin (Arnason^1985^; Belles et al. 1985; Shields et al. 2008; Yencho et al. 1994). Disk assays can also be used on mites (Adesanya et al. 2018; Kerguelen and Hoddle 1999), thrips (Visschers et al. 2018), aphids (Kloth^2015^), whiteflies (Firdaus et al. 2012), or even fungi (Perochon and Doohan 2016) by monitoring visual changes related to the damaged areas of the leaves (Visschers et al. 2018). While disk assays have drawbacks such as the damage inflicted to the tissue, several studies indicate that resistance scores obtained using this approach are comparable to those completed on detached or attached leaves (Visschers et al. 2018).

So far, very few systems have been described to perform such tests on a large number of insects at once. Most experimental setups used image analysis to measure leaf disk area consumed by few larvae either visually (Jones and Coleman 1988), or digitally (Alchanatis et al. 2000; Escoubas et al. 1993), and very few systems allow to track feeding activities over time (Ji et al. 2017; Rowley and Hanson 2007; Rowley et al. 2003). These approaches are well suited to laboratory investigations on small scale series, but handle a limited number of repetitions and often make use of specialized and expensive hardware.

Here, we describe a fast and reliable testing protocol that can be performed using a minimum of hardware components, to measure the time course of the consumption of leaf disks by larvae. Our approach combines three elements (i) a novel and flexible feeding bioassay whereby the consumption of 150 larvae can be followed across several days with a webcam capturing time-lapse images, (ii) a computer program to detect and analyze changes in the surface of leaf disks within an array of cages, and (iii) a new statistical approach to compare the time course of larval feeding activities. As a test case, we monitored second instar European corn borer (ECB) larvae feeding on maize leaf disks treated with different concentrations of NeemAzal and quinine. NeemAzal® is a commercial extract containing mainly azadirachtin, which is considered as antifeedant for several insect species (Schmutterer 1988) including ECB (Arnason et al. 1985; Meisner et al. 1986), but which is also exhibiting insecticide activities (Bezzar-Bendjazia et al. 2017; Isman et al. 1990; Mordue and Blackwell 1993). Quinine is an alkaloid is found in the bark of *Cinchona* and *Remijia* trees (Ruiz-Mesia et al. 2005). This compound is best known as an antimalarial agent and is used at low doses for its bitter taste to humans. It is also “bitter” (French et al. 2015) to Diptera (Meunier et al. 2003), Hymenoptera (Iacovone et al. 2015; Wright et al. 2010), including several Lepidoptera (Minnich 1921; Ramaswamy et al. 1992; Salloum et al. 2011; Shikano et al. 2010), but its effect on ECB larvae is unknown.

## Materials and Methods

### Insect Rearing

*Ostrinia nubilalis* Hübner eggs were obtained from Bioline AgroSciences (France). After eclosion, larvae were maintained in Petri dishes containing an artificial diet (1.32 l water, 27 g agar powder, 224 g corn flour, 60 g dried yeast, 56 g wheat germ, 12 g L-ascorbic acid, 4 g vitamin mixture and minerals (Ref. 0,155,200, D.Plantes Laboratoire, France), 0.8 g chlortetracycline, 2 g hydroxybenzoic acid methyl, 1.6 g sorbic acid, and 4 g benzoic acid), under 16:8 (light: dark) photoperiod at 70% humidity and 26 °C. Second instar larvae (10 days old) were used for the feeding bioassays. We selected individuals which were moving actively and of similar size. These larvae were not starved before the experiment.

### Maize Plants

Seeds from the maize inbred line MBS847_NLN14 were obtained from Saclay’s Divergent Selection Experiments (Aguirre‐Liguori et al. 2019). Seeds were pre-germinated in sprouting trays before being transferred into individual pots (4 l) containing Jiffy® premium substrate. Plants were grown in a greenhouse at 16 h light: 8 h darkness with a temperature between 21–24 °C and 70% relative humidity. Mature leaves of the same leaf rank (rank 8) and from plants at the same development stage, were selected and punched onsite to collect about ten 1 cm diameter disk per leaf, so that the experiment was not destructive to the plants.

### Recording System

**Fig. 1:**
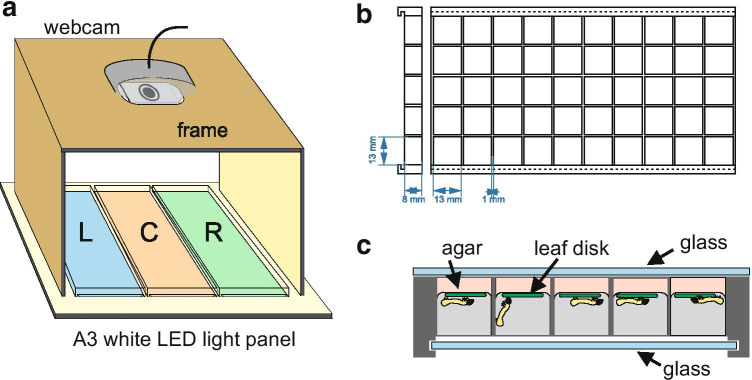
Diagram of the feeding bioassay. **a**. One feeding bioassay setup corresponds to one batch, includes three 50-cages plates numbered L (left), C (center), R (right), and a webcam connected to a computer through a USB port. A white LED lightning board illuminates the plates from underneath **b.** Diagram of a 3D printed plate with 50 cages (13 × 13 × 8 mm) viewed from above and from the side. Longitudinal grooves are present on one face to slide in a glass plate. The other side’s glass plate is placed close to the cages frame and maintained to it with rubber bands. **c.** Profile view of one plate covered with two transparent glasses and showing larvae feeding on leaf disks. A 3–5 mm layer of 1% agar is first deposited on the bottom of the cage, and 1 cm diameter leaf disks freshly punched from a plant are deposited on the agar. Then, the larvae are introduced into the individual cages row by row and enclosed in their cages by sliding a second glass plate over the filled cages. The system is then placed upside down on the light panel so that the camera is able to monitor the disks through the layer of agar with little interference from the larvae crawling inside the cages

Each plate was designed as an array of 10 × 5 individual cages (13 × 13 × 11 mm), separated by 1 mm walls (Sanane et al. 2020b). The bottom of the plate was designed with two grooves to slide in a glass plate cut to the corresponding dimensions. Each cage of the plate was filled up to 2–3 mm with a 1% agar solution to maintain leaf disk moisture during the experiment. One leaf disk and one larva (10 days old) were added to each cage. The top of the plate was then covered with a second glass plate and maintained in place with two rubber bands, to prevent larvae to escape (Fig. 1). A webcam (Logitech HD C920) was placed on a stand made from MDF (Medium Density Fiberboard) cut by laser cutting (Fig. 1). The plates were deposited upside down (so that caterpillars would not obscure the view of the leaf disks) over a white light panel (A3; white LEDs 4000 K; Display Concept, Brussels, Belgium). Each bioassay was done by running two such recording systems at a time, thus simultaneously monitoring six plates with two webcams, but more cameras can be added if necessary (a standard PC computer could run 4 cameras at a time).

The plates were designed with AutoCAD software and 3D printed (Ultimaker 2 +) using a white PLA (polylactic acid) filament (RS Stock No. 134–8192), at a resolution of 0.6 mm. The cost of one system (including the webcam, the LED panel, and the plates) is less than 400 $ US. Instructions for building this experimental system are freely available on a dataverse (Sanane et al. 2020b).

In this experiment, two to four **w**ebcams (Logitech C920 HD Pro) were connected to the USB ports of a PC computer. Time-lapse images were taken every minute from each camera by an open-source video surveillance software (Vision GS BE 3.1.0.4) as 1920 × 1080 pixels jpg files and stored to a disk.

### Experimental Design

The leaf disks were directly deposited into the individual cages with 1% agarose gel after being sampled on the plants to avoid dehydration and tested same day with the larvae. The bioassays consisted in measuring the consumption of the larvae in the absence or in the presence of an antifeedant: NeemAzal®-T/S (Andermatt, France) that contains azadirachtin A, and an alkaloid, quinine hydrochloride (Sigma Aldrich; CAS number: 6119–47-7). Each leaf disk received 10 µl of the substance diluted in distilled water and was kept 15 to 20 min at ambient temperature to let the solution evaporate before the experiment started. Five concentrations were used for NeemAzal and quinine (Table 1). One larva (second instar) was deposited in each cage and allowed to feed for 48 h at ambient temperature. One treatment (i.e. one specific concentration of the product) was tested in two plates. For each product, we conducted two bioassays/batches that started on two consecutive days (Table 1). In each batch, treatments were randomly assigned to the plates. As we tested two antifeedant products, this experimental design led to the acquisition of 1200 feeding curves (2 products * 6 treatments * 2 replicates * 50 leaf disks).

**Table 1:** Experimental design

Antifeedant	Concentration	Batch			
		2019–06-18	2019–06-19	2019–06-27	2019–06-28
Control		Cam02-L	Cam02-C	Cam01-L	Cam03-C
NeemAzal (mM)	0.01	Cam01-L	Cam01-R		
	0.1	Cam01-R	Cam01-C		
	1	Cam02-C	Cam02-L		
	3	Cam02-R	Cam02-R		
	10	Cam01-C	Cam01-L		
Quinine (mM)	0.01			Cam02-L	Cam02-R
	0.1			Cam01-C	Cam01-R
	1			Cam02-R	Cam02-L
	10			Cam01-R	Cam01-L
	100			Cam02-C	Cam02-C

The experiments were performed over 4 days, using 2 cameras. Each batch corresponds to an experiment performed on the same day with six different plates. One plate in a batch corresponds to one treatment. The plates are identified by the camera number and their position in the experimental set-up (L = left, C = center, R = right). Neem corresponds to NeemAzal treatment and Quin to Quinine treatment

### Image Analysis

Image analysis of the stacks was performed semi-automatically, with the help of custom plugins written with Java using the Eclipse programming environment (http://www.eclipse.org), and hosted by the bio-imaging open-source program ICY (Chaumont et al. 2012).

**Fig. 2:**
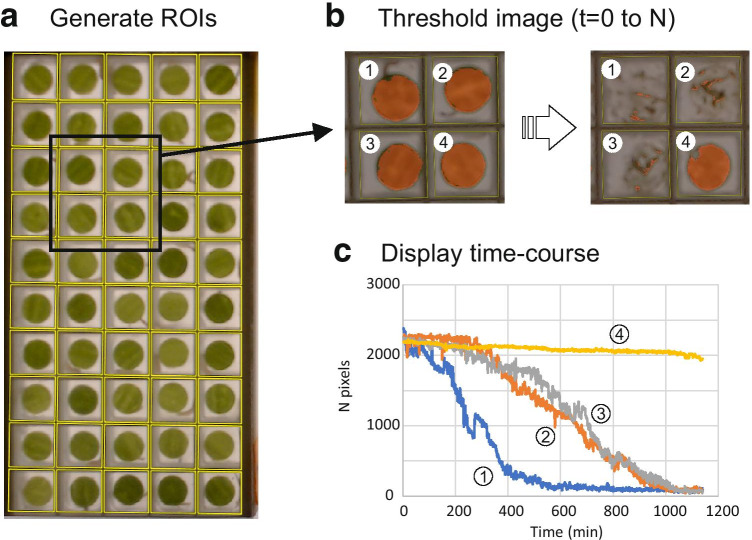
Image analysis workflow. **a**. The first step of the analysis is to define an array of regions of interest (ROIs) defining the limit of each cage. This is done interactively under ICY, using a custom plugin RoitoRoiArray. On this picture, only one plate is displayed with the ROIs in place (yellow). **b.** The second step is to choose a filter procedure and a threshold adapted to detect the leaf disks, based on the intensity of the filtered image (obtained by combining the 3 color planes) or based upon a color space defined by points sampled by the user over the image. The corresponding area is overlayed in red over the original image. On this figure, we have selected and zoomed over 4 cages at the beginning and at the end of an experiment. The whole stack of images is then analyzed to measure the number of corresponding pixels in each ROI. The filtering and measuring (and data export) are done interactively under ICY, using a custom plugin AreaTrack **c.** Evolution of the surface of the 4 disks represented in Fig. 2b

Regions of interest (ROIs) were first defined to identify each cage or disk with a plugin called RoitoRoiArray (http://icy.bioimageanalysis.org/plugin/roitoarray/). This cannot be done fully automatically because there are always minute differences of position and lightning between experiments. We used a simple principle which is that the pixels of lines parallel to the borders of a plate are less variable when the lines run over the cage limits (where pixels have roughly the same color) than when they cross the cages (where pixels are grey then green or black). Therefore, starting from a parallelogram enclosing a plate drawn by the user, this program could find the number of cages and propose lines displayed over the cage limits. These lines, eventually edited by the user, were used to generate an array of ROIs, each with a unique descriptor (name and ID), and saved into an XML file for further reference and use (Fig. 2a, b).

A second plugin, Areatrack (http://icy.bioimageanalysis.org/plugin/areatrack/), was used to analyze the images stacks, first to define an image filter to detect the leaves and to evaluate their surface (Fig. 2c, d). Each image of the stack was filtered and binarized to keep only pixels corresponding to the leaf areas. As considerable variation of lightning and colour of the leaf disks occurred, two detection strategies were implemented. The first was to apply a filter to the RGB image (R = red, G = green, B = blue) using simple arithmetic filters comparing RGB intensities. This was done either by isolating one color plane (R, G or B), or by calculating an arithmetic combination of these planes such as: 2R-(G + B), 2G-(R + B), 2B-(R + G), (R + G + B)/3, or by converting the image into one component of the HSB space (H = hue, S = saturation, B = brightness). Most of the time, the 2G-(R + B) filter with a threshold of 35 gave satisfactory results. Although this approach worked well in many cases, some experiments could not be analysed this way, because the leaf colours and transparency were such that none of these filters allowed enough contrast between the leaves and the background over the whole image. We introduced another approach, to filter all pixels whose colour fell were close to an array of reference colours. The reference colours were defined by the user who add colours by pointing at pixels on the screen within the leaf disks. To filter the image, we defined two measures of colour distance L1 and L2. Distance L1 between pixel i and colour k was defined in the RGB space as:

$$L1=\left|{R}_{i}-{R}_{k}\right|+\left|{G}_{i}-{G}_{k}\right|+\left|{B}_{i}-{B}_{k}\right|$$

where R = red, G = green, B = blue, i = pixel index (1 to the number of pixels of the image), and k = reference color index (1 to the number of reference colours).

Distance L2 was defined as:

$$L2=\sqrt{\left({\left({R}_{i}-{R}_{k}\right)}^{2}+{\left({G}_{i}-{G}_{k}\right)}^{2}+{\left({B}_{i}-{B}_{k}\right)}^{2}\right)}$$

Each pixel of the image was compared with each “reference” colour, and this pixel was accepted if its distance from one of the references was lower than a threshold defined by the user. The user could add or remove reference colours, adjust the distance type and a threshold value, and directly visualize the result of this custom filter as an overlay over the original images. As a rule of thumb, 3–5 reference colours were usually enough to build a filter that could run across all leaf disks, using distance L2. Given the flexibility of this approach, this method was used most of the time.

Although some recordings were performed on longer time periods, this paper reports analyses only made over a 48 h, corresponding to stacks of 2,880 images per plate, each image weighting about 390 Kb as jpg files. On our computer, analyzing a stack of 1142 pictures took 120 s with a regular filter (2G-R-B), 144 s with a color filter with 3 reference colors (L2 = 25), and 186 s with 8 reference colours (L2 = 10). To shorten this disk access time, we implemented a procedure to fetch data in advance from the disk. We also included procedures to subsample the data, for example, to analyze only one image under 10 or under 60, in case users did not need a 1-min precision.

Once ROIs (to define the cage limits) and the colour filters were properly defined (to extract the leaves), the entire set of data could be analyzed. The surface of the leaves was estimated by counting the number of pixels over the threshold in each ROI and in each image. The resulting measures were exported as an Excel file, including (i) the raw measure of surface for each ROI (i.e. the number of pixels over the threshold in each cage), (ii) a running average of these measures, computed over consecutive images (generally n = 10) and (iii) a running median.

### Data Analysis

Series of R scripts were written for further data processing and statistical analyses. The scripts, along with the raw data and documentation are available in a dataverse (Sanane et al. 2020a).

### Metadata

The raw data files were stored using laboratory storage facilities on specific directories, names according to the date of the experiment, and the camera number. Excel files were first converted into Csv filenames and edited to contain all the information about the experiment: date, camera ID, plate ID, insect population, plant genotype, plant environment, plant coordinates, substance name, substance concentration. After image analysis, all csv files to be analyzed together were copied under a single sub-directory.

### Standardization

First, the leaf consumption of each larva through time was plotted and displayed by groups of 50 curves, which is the number of cages within a plate. Cages that produced abnormal plots (with a sudden change of the area caused for example by a leaf rolling onto itself or pushed away by the larva), were either re-analyzed using a different set of filters or removed from the analysis. After this manual cleaning, we standardized the data by dividing the leaf area at time t by the leaf area at time *t0*. Hence, the basic measure became the fraction of intact leaf disk from each cage at each time-point. We defined *tmax* = 2500 min (40 h), and only retained the data taken before *tmax*.

### Data Clustering

The whole dataset, comprising the experiments performed with NeemAzal, quinine, and the control (4 batches, 24 plates, 1200 cages corresponding to 1200 consumption curves), was used to run the unsupervised clustering algorithm SOTA (Herrero et al. 2001) on the individual curves to obtain 14 clusters. The total number of clusters was empirically chosen to avoid having clusters containing only a couple of curves and proved to be robust over the different experiments.

### Typology of Feeding Behavior

Each curve was characterized by the time after which 20%, 50% and 80% of the leaf disk were consumed, respectively noted t20, t50, t80, and the fraction of the leaf disk consumed at *tmax*. When less than 20%, 50%, or 80% of the leaf disk was consumed at *tmax*, the corresponding variable was given the value of *tmax*. Then, each cluster was characterized by the median t20, t50, t80, and total fraction consumed, using the values of the curves belonging to the cluster (Fig. 3.2). We then arranged the 14 clusters into 6 groups based on their median values for t20, t50, t80, and total fraction consumed using the K-means algorithm (Hartigan and Wong 1979). Each group corresponded to a different feeding behaviour named by a letter from A to F (Fig. 3.2). All the curves belonging to the same behavioural group were assigned the same letter and are referred as “behavioural type” below and in the results section.

### Data Transformation

At the end of the analysis, each cage l, corresponding to treatment i, batch j, and plate k was characterized by the feeding behavioural type (A to F) of the group to which it belongs. Hence, the observations of a single cage can be summarized into a vector of zeros and ones ${Z}_{ijkl}=\left({Z}_{ijkl}^{1},\dots ,{Z}_{ijkl}^{w},\dots ,{Z}_{ijkl}^{W}\right)$, (1) where w represents one feeding behaviour, with w in {A, B, C, D, E, F}, and ${Z}_{ijkl}^{w}=1$ if the observed feeding behaviour is w, and ${Z}_{ijkl}^{w}=0$ if else. For a given cage subscripted by *ijkl*, ${\sum }_{w=1}^{W}{Z}_{ijkl}^{w}=1$

### Statistical Analysis

A separate analysis was conducted for each product (NeemAzal and Quinine). ${Z}_{ijkl}$ is the result of one multinomial sampling in

1 $${Z}_{ijkl}\equiv M\left(1,{p}_{ijk}^{1},...,{p}_{ijk}^{w},...,{p}_{ijk}^{W}\right)$$

where ${p}_{ijk}^{w}=P\left({Z}_{ijkl}^{w}=w\right)$ is the probability that a single observation falls into the feeding behaviour *w*. We used the logistic multinomial regression (Hartigan and Wong 1979) to estimate the probabilities.

For a given product, the full model is

$$log\left(\frac{{p}_{ijk}^{w}}{{p}_{ijk}^{W}}\right)={{\mu }}^{w}+{\alpha }_{i}^{w}+{\beta }_{j}^{w}+{\gamma }_{ik}^{w}$$

where ${\alpha }_{i}^{w}$ is the treatment effect, ${\beta }_{j}^{w}$ is the batch effect, and ${\gamma }_{ik}^{w}$ is the interaction between plate and treatment effects.

The experimental setting was highly imbalanced. For example, only control plates from the two quinine batches were added to the NeemAzal experiment, so that it was not possible to test for the batch effect. Similarly, because we used only two plates for each treatment (except for the controls), interactions between plates and treatments cannot be estimated. We, therefore, used the submodel (2) to infer the effects of treatments using:

2 $$log\left(\frac{{p}_{ijk}^{w}}{{p}_{ijk}^{W}}\right)={{\mu }}^{w}+{\alpha }_{i}^{w}$$

where ${\mu }^{w}$ is the average proportion of the feeding behaviour w, ${\alpha }_{i}^{w}$ is the effect of treatment i, and W is the reference feeding behaviour. Submodel (2) was compared to a model where the differences between the observations were only due to the plates, whatever the treatment

3 $$log\left(\frac{{p}_{ijk}^{w}}{{p}_{ijk}^{W}}\right)={{\mu }}^{w}+{\gamma }_{k}^{w}$$

Models (2) and (3) were compared using the Akaike Information Criterion (AIC) (Akaike 1998). In the two experiments, model (2) was chosen by the AIC criterion, meaning that probabilities that one cage falls in one given behaviour group rather than another depend more on the treatment (product concentration) than the plate it belonged to.

The multinomial regression (2) provided an estimation of the probabilities associated with each treatment, using

$${\widehat{p}}_{i}^{w}=\frac{{e}^{{\widehat{\mu }}^{w}+{\widehat{\alpha }}_{i}^{w}}}{1+{\sum }_{v}{e}^{{\widehat{\mu }}^{v}+{\widehat{\alpha }}_{i}^{v}}}$$

that were used for the graphical representations (Fig. 4).

A Wald test was performed to compare the treatments (Davidson and Mackinnon 1993). Contrasts between two treatments i and i’ are computed as the differences ${\alpha }_{i}^{w}-{\alpha }_{i\text{'}}^{w}$. A positive value of the contrast means that the proportion of feeding type w relative to feeding type W is greater in treatment i than in treatment i’. With multinomial regression, the choice of the reference is tricky. The best reference is the category where the observations are equally distributed between the treatments. We chose the feeding type F as the reference for the quinine experiment, and the feeding behaviour E for the NeemAzal experiment.

## Results

### Feeding Activities

During these experiments, larvae exhibited different behaviours, ranging from immediate feeding until the whole disk was consumed to not feeding at all. We defined feeding types based upon the kinetics of their feeding. First, by considering the time course of the feeding of each of the larvae tested, we defined a typology of possible behaviours exhibited in the whole dataset. Second, we compared the distribution of behavioural types between treatments of the same product.

### Feeding Types

**Fig. 3:**
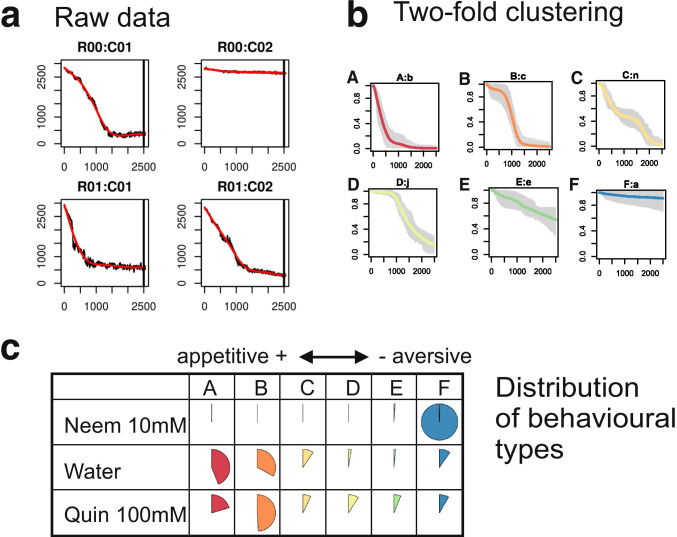
Data analysis workflow under R. **a**. Raw data processing. The surfaces measured in pixels are exported as a table (column = ROI, row = time) and displayed as an array curves using the same disposition as the plates. Here, 4 of such curves are displayed corresponding to the raw experimental data of Fig. 2c, with time in abscissa, and the number of pixels as ordinates. Each figure displays the raw data (black curve) and a filtered curve over it (in red). These curves are printed to pdf files to let the user check visually the quality of the results. The tables are then transposed (ROI = row, time = column) and further analyzed with R procedures. **b.** Two-fold clustering. First, SOTA classification results into 14 clusters. Each cluster is represented by a vignette showing the median consumption curve (color line) and the variations around the median at each time point (grey area). Second, SOTA clusters are grouped into six types using the median value of characteristic times t20, t50, t80 and total consumption representing the clusters. At the end, the consumption curve of each cage is attributed a behavioural type, between A and F. **c.** Results: distribution of behavioural types for each treatment. As an example, we show here the distribution of behavioural types in three treatments: NeemAzal 10 mM, Water (control), and Quinine 100 mM. The frequency of types (A to F) in each category (column: type, row: stimulus) is represented as a pie chart. Each row sums to 1

According to this approach, the individual consumption curves can be classified into 6 different types using the SOTA algorithm, which is particularly adapted to the classification of temporal data. By running this method on the 1200 consumption curves, we obtained 14 different clusters of curves (Fig. 3b). As shown in Fig. 3b, variations of individual behaviours around the cluster median are moderate during the first 24 h. The average within-cluster coefficient of variation ranges from 3% after one hour to 25% after 16 h. It increases up to 125% at the end of the experiment. Each cluster was further characterized by traits like the time to consume 20%, 50%, or 80% of the leaf disk (noted t20, t50, t80), as well as the total consumption at the end of the experiment. The median values of these traits were used to reduce the 14 clusters into 6 behavioural types (Fig. 3b):

- A-type larvae immediately start to feed (low t20), consume fast (low t50, t80), and finish the leaf disk before the end of the experiment.
- B-type larvae tend to wait before consuming (high t20) but consume fast after the waiting time and generally consume all the leaf disk.
- C-type larvae consume fast at the beginning (low t20) but reduce their consumption rate through time, resulting in low t50 but high t80.
- D-type larvae show a behaviour intermediate between B and C, with high t20 and high t50.
- E-type larvae are reluctant to start feeding, showing high t20, t50, and t80, but achieving a significant consumption at the end.
- F-type larvae do not consume the leaf disk.

Using this typology, each curve can be assigned to a single behavioural type (A to F), thus reducing the comparison between treatments to comparisons between the proportions of each behavioural type within each treatment (Fig. 3.3). Figure 3.3 shows that when maize leaves are treated with water, most larvae can be classified as A or B type and consume the maize disk rapidly. The treatment with 100 mM quinine gives almost the same results. However, when maize leaves are treated with 10 mM NeemAzal, most of the larvae do not consume the leaves and are classified into the F type.

Altogether, this analysis revealed the diversity of behavioural responses of ECB larvae when feeding on maize leaf disks. Notice that even with the water control treatment, a fraction of the larvae did not consume the leaf disk.

### Bioassays Confirm Neemazal as an Antifeedant

**Fig. 4:**
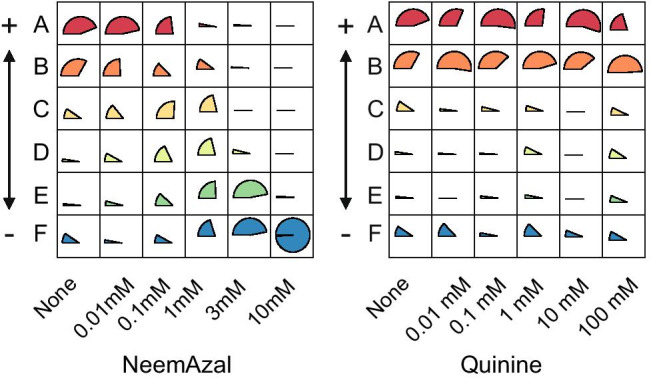
Distribution of behavioural types for the different treatments. For each treatment (column), the estimated probability of each behavioural type is represented as a pie. Each column sums to one. **a**. Neemazal treatments. Neemazal concentrations are reported as legend to each column. « None» corresponds to the control with water. **b**. Quinine treatments. Quinine concentrations are indicated in the column names. « None» corresponds to the control with water. The colour gradient corresponds to appetitive (red) to aversive (blue)

After using the whole dataset to attribute a behavioural type to each individual (Fig. 3), the effect of each treatment (i.e. antifeedant concentration) was analyzed separately for each antifeedant product by a multinomial regression (see Methods). Each analysis included the data of each dose of antifeedant and the controls, and resulted in the estimations of the probability distribution among behavioural types for each concentration of each product that is represented in Fig. 4. A Wald test was used to test the significance of the comparisons between treatments (SI-Table 1 and SI-Table 2).

In the NeemAzal bioassays, an increasing concentration of the product was associated with a decreasing proportion of A and B larvae and an increasing proportion of E and F larvae, while intermediate concentrations corresponded to a higher proportion of C, D, E larvae (Fig. 4a). Without antifeedant, most individuals were classified as A and B, *e.g.* the larvae started feeding immediately (A-type) or waited a short time (B-type) but then quickly consumed the whole leaf disk. For intermediate concentrations of the antifeedant, a large fraction of the larvae started consuming at a lower rate (C-type) or waited and consumed slowly (D-type). Most of them did not finish eating their leaf disk. With 10 mM NeemAzal, 98% of the larvae were classified as F type, which corresponds to the absence of consumption.

These results are confirmed by contrast estimates relative to type E (SI-Table 1). A positive value of the contrast between treatments i and i’ means that the proportion of type w (w being A, B, C, D, or F) relative to type E is greater in treatment i than in treatment i’. All contrasts between 10 mM NeemAzal and lower concentrations are significantly negative for types A, C, D, and significantly positive for type F, indicating a strong deficit of types A, C, D and a strong excess of type F in the 10 mM NeemAzal treatment. Notice that for the B type, the comparisons lead to negative but non-significant contrasts. This may indicate a lack of power of the experimental design. NeemAzal treatments 0.1 mM and 0.01 mM are generally non-significantly different from the control, except for types A, B C. The negative value of the significant contrasts always indicates an excess of types A, C, and C in the control. Altogether, these analyses indicate that ECB larvae detect NeemAzal as an antifeedant, and change their feeding behaviour according to the concentration of the product.

In the quinine bioassay, we hardly saw any effect of the antifeedant concentration on the distribution of behavioural types, except for a slight excess of feeding types C, D, E in the 100 mM treatment as compared to 10 mM (Fig. 4b). This result is supported by the contrasts that were hardly significant (SI-table 2). Even when leaf disks were treated with 100 mM quinine, the proportion of larvae showing an A/B behaviour was still elevated. This indicates that quinine, when diluted with water, has no effect on larvae feeding and that most larvae consume the leaf disk entirely although a fraction do not start to consume immediately.

## Discussion

In this paper, we established a new data collection method to evaluate the consumption of Lepidopteran larvae of maize leaf disks. As a test case, we analysed how larvae modulate their feeding activities when leaf disks are treated with NeemAzal®, and quinine. NeemAzal has a concentration-dependent impact on the distribution of consumption curves (Fig. 4a). Our results confirm that azadirachtin is an antifeedant for ECB larvae (Arnason et al. 1985; Meisner et al. 1986). Although this compound is noted as having insecticidal activities on ECB larvae, we did not observe much mortality over the duration of our tests at the doses tested. Contrary to our expectations, ECB larvae did not avoid quinine and may even like it. Although this alkaloid is avoided by many animals belonging to different genera, scattered observations in the literature reported the absence of bitterness of quinine in a few species like another Lepidoptera, *Cydia pomonella* (Pszczolkowski 2017), and in two mantid and two spider species (Mebs et al. 2019). While this observation is new for *O. nubilalis*, it remains to be tested whether the lack of response to quinine is species- or stage-specific.

While the leaf-disks method is commonly used to screen for plant resistance or to evaluate antifeedant compounds, this approach has the drawback of using an excised plant tissue, which may desiccate, and which is injured. Hence, most experimenters place the disks over agar to limit desiccation during the experiment (Escoubas et al. 1993; Little et al. 2017). To limit these effects, we collected the leaf disks in the field on the day of the experiment, placing them immediately in the cages together with 1% agarose, and sandwiched them between two glass plates as shown in Fig. 1, before bringing them back to the laboratory. Despite these precautions, we observed in some experiments, that some leaf disks changed their colour over the course of the experiment, becoming yellowish or paler. Since leaf disks are mechanically excised, the tissue is wounded especially along the border of the disk (Jones and Coleman 1988). However, several studies found no differences between detached/attached leaves and leaf disc assays (Visschers et al. 2018). Notice that the use of excised leaf disk allows a physical separation between the production of the plants and the location where the pest insects are held, and therefore minimizes the risks of contamination of the plant production site. Nevertheless, because of these limitations, the results obtained with leaf disks ought to be considered with caution.

While videographic analyses were proposed earlier to measure leaf consumption from a limited number of larvae (Ji et al. 2017; Rowley and Hanson 2007; Rowley et al. 2003), our system is the first to run on such a large sample of insects and to analyze the time course of the feeding. Using 3D-printed cages and simple equipment, a common computer, and a webcam, we were able to follow the feeding activities and movements of 150 larvae during several days. In addition, our system was built with on-the-shelf readily available elements and can be duplicated and adapted to different situations. Actually, the most limiting factor of our approach is the time necessary to collect the larvae and to introduce them into individual cages. In our laboratory, a single operator could start two batches of 150 larvae a day. For example, the whole NeemAzal experiment (600 larvae) presented here was realized in two days, using four feeding bioassays devices in parallel.

Classical consumption tests usually rely upon comparing feeding after fixed interval(s) of time (Arnason et al. 1985; Clark et al. 2014; Huang et al. 2003; Isman et al. 1990; Menezes et al. 2005; O'Neal et al. 2002). The consumption can be estimated from images taken before and after or by any other means, for example by a gravimetric method (Clark et al. 2014). With this protocol, the measuring time needs to be defined a priori as the measure is taken only one time, for example, 1 h, 10 h, or 24 h after the start of the experiment. One way to adjust the observation period to the feeding activities of the insects is to wait until enough feeding has occurred in the control (for example 50% or 90% consumption). Although such methods are sufficient to detect antifeedant activities, they do not consider the time course of the feeding activities and do not allow, for example, to distinguish between an initial repellent effect that can be overcome later from a pure antifeedant effect.

We introduced a new rigorous statistical approach to analyze and compare the time course of feeding activities of individual larvae. With the approach developed here, we can characterize properly the different feeding strategies adopted by large cohorts of insects confronted with the same situation. While the classical approach and our approach may globally obtain comparable results, we will be able to better characterize whether plant resistance factors or externally applied chemicals have an immediate sensory effect or if they are the consequence of post-ingestive effects.

Insect feeding bioassays are of importance for plant health and particularly plant breeding programs. They allow evaluation of plant resistance against insects with chewing mouthparts by measuring the area of tissues consumed. The system described is affordable and can be adapted to different insects and plants. As it opens up the possibility to test a large number of insects at once, it may become an essential tool to screen for plant resistance to pests, to find rare individuals who are resistant to such plants or insecticides, and but also to study the phenotypic variability of feeding in natural populations of insects.

## Supplementary Information

Below is the link to the electronic supplementary material.

Supplementary file1 (PDF 125 KB)

## Data Availability

The maize lines used in this paper are available upon request from INRAE le Moulon. The experimental data images and measures are available upon request from the corresponding author.

## Code Availability (Software Application Or Custom Code)

The R scripts described in this manuscript as well as the 3D schematics are available on dataverse described in the text, and the Java plugins from Icy website.
