# Supplementary material for: High-Throughput Feeding Bioassay for Lepidoptera Larvae
Source: J Chem Ecol. 2021 Jul 31;47(7):642–52. doi: 10.1007/s10886-021-01290-x (PMC8346434; doi:10.1007/s10886-021-01290-x)
Supplement: Supplementary file 1 — Supplementary file1 (PDF 125 KB) [file 10886_2021_1290_MOESM1_ESM.pdf]

Supplementary information

SI_Table 1: Contrasts between the different concentrations of NeemAzal treatments within each typology of feeding behaviour. Contrasts are expressed on the logit scale, relative to the reference feeding behavioural category E. For each contrast and each behavioural type, the levels of significance is indicated by the colour filling. Dark gray = significant at the 1‰ risk level.1. Light grey = significant at the 1% risk level. White = non-significant at the 1% risk level.

Contrast	A	B	C	D	F
neem_10-3mM	-10,84	-8,32	2,17	-12,11	4,59
neem_10-1mM	-12,61	-11,29	-15,31	-14,44	4,78
nem_10-0,1mM	-15,34	-12,25	-16,22	-14,98	5,00
neem_10-0,01mM	-17,13	-13,96	-16,70	-15,27	4,88
neem_10mM-Control	-18,07	-15,26	-17,29	-15,08	2,75
neem_3-1mM	-1,77	-2,97	-17,48	-2,33	0,18
neem_3-0,1mM	-4,50	-3,93	-18,39	-2,87	0,41
neem_3-0,01mM	-6,29	-5,64	-18,87	-3,16	0,29
neem_3mM-Control	-7,23	-6,94	-19,46	-2,97	-1,85
neem_1-0,1mM	-2,73	-0,96	-0,91	-0,54	0,22
neem_1-0,01mM	-4,52	-2,67	-1,39	-0,83	0,11
neem_1mM-Control	-5,46	-3,97	-1,98	-0,64	-2,03
neem_0,1-0,01mM	-1,79	-1,71	-0,48	-0,29	-0,12
neem_0,1mM-Contol	-2,73	-3,01	-1,07	-0,10	-2,25
neem_0,01mM-Control	-0,94	-1,30	-0,59	0,18	-2,13

SI-Table 2: Contrasts between the different concentrations of Quinine treatments within each typology of feeding behaviour. Contrasts are expressed on the logit scale, relative to the reference behavioural type F. For each contrast and each behavioural type, the levels of significance is indicated by the colour filling. Dark gray = significant at the 1‰ risk level. Light grey = significant at the 1% risk level. White = non-significant at the 1% risk level.

	A	B	C	D	E
quin_100-10mM	-1,3	-0,08	16,06	16,47	15,12
quin_100-1mM	0,14	0,49	0,742	0,657	1,099
quin_100-0,1mM	-1,94	-0,75	-0,42	1,216	0,118
quin_100-0,01mM	0,05	0,41	1,738	2,683	15,17
quin_100mM-Control	-0,62	0,55	-0,13	1,453	1,558
quin_10-1mM	1,44	0,57	-15,3	-15,8	-14
quin_10-0,1mM	-0,64	-0,67	-16,5	-15,3	-15
quin_10-0,01mM	1,35	0,49	-14,3	-13,8	0,054
quin_10mM-Control	0,68	0,63	-16,2	-15	-13,6
quin_1-0,1mM	-2,08	-1,24	-1,16	0,559	-0,98
quin_1-0,01mM	-0,1	-0,09	0,996	2,026	14,07
quin_1mM-Control	-0,76	0,05	-0,88	0,796	0,46
quin_0,1-0,01mM	1,98	1,15	2,16	1,467	15,05
quin_0,1mM-Control	1,32	1,29	0,288	0,237	1,441
quin_0,01mM-Control	-0,66	0,14	-1,87	-1,23	-13,6
